# Estimated Energy Expenditure in Youth While Playing Active Video Games: A Systematic Review

**DOI:** 10.3390/sports12020039

**Published:** 2024-01-25

**Authors:** Cíntia França, Sadaf Ashraf, Francisco Santos, Mara Dionísio, Andreas Ihle, Adilson Marques, Marcelo de Maio Nascimento, Élvio Rúbio Gouveia

**Affiliations:** 1 Department of Physical Education and Sport, University of Madeira, 9020-105 Funchal, Portugal; cintia.franca@staff.uma.pt (C.F.); sadaf.ashraf0031@gmail.com (S.A.); francisco191santos@gmail.com (F.S.); 2 LARSYS, Interactive Technologies Institute, 9020-105 Funchal, Portugal; mara.dionisio@staff.uma.pt; 3 Research Center in Sports Sciences, Health Sciences and Human Development (CIDESD), 5000-801 Vila Real, Portugal; 4 Faculty of Sport Sciences and Physical Education, CIDAF, University of Coimbra, 3040-248 Coimbra, Portugal; 5 Faculty of Exact Sciences and Engineering, University of Madeira, 9020-105 Funchal, Portugal; 6 Department of Psychology, University of Geneva, 1227 Carouge, Switzerland; andreas.ihle@unige.ch; 7 Center for the Interdisciplinary Study of Gerontology and Vulnerability, University of Geneva, 1227 Carouge, Switzerland; 8 Swiss Center of Expertise in Life Course Research LIVES, 1227 Carouge, Switzerland; amarques@fmh.ulisboa.pt (A.M.); marcelo.nascimento@univasf.edu.br (M.d.M.N.); 9 CIPER, Faculty of Human Kinetics, University of Lisbon, Cruz Quebrada, 1499-002 Lisboa, Portugal; 10 Environmental Health Institute (ISAMB), Faculty of Medicine, University of Lisbon, 1649-020 Lisbon, Portugal; 11 Department of Physical Education, Federal University of Vale do São Francisco, Petrolina 56304-917, Brazil

**Keywords:** exergames, physical activity, heart rate, accelerometry, children, adolescent

## Abstract

Sedentary behavior and inadequate energy expenditure are serious global public health concerns among youngsters. The exponential growth in technology emerges as a valuable opportunity to foster physical activity, particularly through active video games. We performed a systematic review following the Preferred Reporting Items for Systematic Reviews and Meta-Analyses guidelines in PubMed, Web of Science, Cochrane, and Scopus to provide a comprehensive view of the literature on energy expenditure levels among adolescents while playing active video games. Among the 574 manuscripts identified at the first screening stage, 23 were retained for analysis. Ten studies were characterized by longitudinal and thirteen by cross-sectional designs. The results showed that short-term active video games elicited energy expenditure values comparable to moderate-intensity physical activity (3–6 METs). However, in intervention programs (with at least six weeks) the results indicate no significant effects of active video games on youngsters’ energy expenditure levels and physical activity profiles between baseline and follow-up assessments. Overall, active video games based on sports and dance were the most used, and boys tended to achieve higher energy expenditure than girls. The diversity of methods implemented limits comparing results and drawing generalized conclusions. However, considering its attractiveness to youth, active video games might emerge as a complementary tool to traditional physical activities promoted in schools and local communities. Details regarding gender differences and contradictory results of longitudinal approaches should be considered in future research based on standardized methods.

## 1. Introduction

Worldwide, it is estimated that nearly 81% of adolescents aged between 11 and 17 years do not meet the recommended physical activity (PA) levels [1]. This is highly concerning since increased time spent in sedentary behavior (SB) and low PA have been associated with overweight and obesity, which represents severe public health problems. According to the literature, obesity is the result of the imbalance between energy intake (EI) and energy expenditure (EE) [2], influenced by physiological, psychological, and environmental factors [3]. Therefore, estimating EE is critical in the global context of non-communicable diseases [4].

In the literature, the doubly labeled water technique is described as the “gold standard” approach to assessing EE [4], which involves elevated costs and does not provide information on PA patterns [5]. PA is any bodily movement elevating the EE above resting levels [6]. Thus, levels of PA are directly related to EE [7]. Therefore, motion sensors, such as accelerometers, are available and accessible tools frequently used to measure PA and estimate EE [3]. 

To address the fight against SB and promote PA levels among children and adolescents, active video games (AVGs) that integrate exercise and gaming entertainments have emerged as an innovative approach [8]. Several investigations based on AVGs have targeted children and adolescents, particularly overweight and/or obese [9,10], and older adults [11,12]. The literature results indicate that AVGs can generate health benefits in physical fitness and weight loss [8,13]. Indeed, previous research has given much attention to the effects of AVGs on weight-related outcomes, with a significant reduction in body weight and body mass index being reported after a PA intervention program based on AVGs [14,15]. Therefore, not surprisingly, AVGs have been seen as an innovative and engaging tool to enhance PA among overweight participants. Particularly for that reason, EE assessment while playing AVGs is crucial to understanding the type of activity intensity that might be achieved. In a systematic review conducted on this topic, the authors reported that EE levels observed ranged between 2.0 and 5.0 metabolic equivalents of tasks (METs) [16]. In another review article, games examined elicited an EE at or above 3 METs, which corresponded to moderate intensity PA [17]. In the previous studies mentioned, the literature search included young participants aged up to 18 [17] or 21 [16] years old, and EE assessments based on indirect calorimetry [17]. Moreover, most of the investigations evaluated were based on cross-sectional analysis deployed in a laboratory context.

However, another important matter to be considered is the differences between genders. Research has shown that girls tend to be less active than boys. Possible justifications for this conclusion are related to girls’ lower participation in organized sports [18], less social support to engage in PA [19], and probably their less perceived enjoyment during Physical Education classes [20]. Thus, intervention programs based on PA tailored according to gender are recommended.

Since AVGs capitalize on youth interest in computerized video games and the need to increase PA [8], AVGs may represent a tool for the promotion of more active lifestyles. Therefore, the present systematic review aims to (1) provide a comprehensive view of the literature on EE levels examined through objective measures among children and adolescents while playing AVGs and (2) summarize the characteristics (i.e., games used, sessions’ duration, instruments used to play) of the studies analyzed. Based on previous literature, the current study aims to clarify the following questions: (1) Which type of PA intensity can be achieved through AVGs?; (2) What are the most common games and instruments used to play?; and (3) Are there differences in EE between boys and girls while playing AVGs? The information gathered through these questions is of great interest for the designing of innovative PA programs that can engage and motivate young populations.

## 2. Materials and Methods

This systematic review followed the Preferred Reporting Items for Systematic Reviews and Meta-Analyses (PRISMA) 2020 guidelines [21]. The PRISMA checklist is available in the supplementary file (Figure S1).

### 2.1. Search Strategy

Four comprehensive electronic databases (PubMed, Web of Science, Cochrane, and Scopus) were screened by three independent authors (C.F., S.A., and F.S.). Primary sources articles published until June of 2023 were considered eligible. The following terms were defined and used to search at the title/abstract level: “serious gam*”, “exergam*”, “active video game”, “child*”, “adolescen*”, “you*”, “energy expenditure”, “metabolic equivalent”, “accelerometer*”, “pedometer*”, “heart rate”, and “activity monitor*” combined with the Boolean operators “OR” and “AND”.

### 2.2. Inclusion Criteria

Inclusion criteria considered were as follows: (a) participants aged between 9 and 17 years from both sexes; (b) studies that assessed EE during AVGs played for at least 30 min; (c) reports of at least one outcome related to EE (light PA-LPA, moderate PA-MPA, vigorous PA-VPA, moderate-to-vigorous PA, metabolic equivalent—MET, heart rate—HR) evaluated through objective measures; and (d) full text written in English, Spanish, and Portuguese, published in the past 20 years (1 January 2023 until 1 July 2023).

### 2.3. Exclusion Criteria

Exclusion criteria considered were as follows: (a) participants with injury, illness, or disabilities; (b) no reports on at least one variable concerning EE; (c) descriptive studies, systematic reviews, protocols, book chapters, conference abstracts, or letter to editors; and (d) studies written in other languages or published before 2003. Regarding the study design, the authors have chosen to focus on experimental studies with a full description of the methods implemented and the results achieved. 

### 2.4. Screening Strategy and Study Selection

All returning documents were compiled into a reference manager (EndNote X20, Thomson Reuters, Philadelphia, PA, USA) for additional analysis after the search completion. After deleting duplicate entries (automatically and manually), potentially eligible studies were independently assessed by three authors (C.F., S.A., and F.S.) to guarantee that articles met the inclusion criteria. Then, the same authors performed a full-text reading for eligible studies. In case of disagreements, a final decision was made by a fourth and more experienced author (E.R.G.) based on the revision of the inclusion/exclusion criteria and the review objectives.

### 2.5. Data Extraction and Harmonization

All authors discussed the criteria for data extraction and harmonization. The following categories were systematically extracted for eligible studies: (1) author name and study year, (2) study’s purpose, (3) sample characteristics (number of participants, age, and sex), (4) instruments and measures, (5) active video games used, number of sessions and program duration, and (6) main findings. 

### 2.6. Study Risk of Bias

The quality assessment was performed using the Effective Public Health Practice Project (EPHPP) [22], and the modified version of the Newcastle-Ottawa Scale (NOS) [23], according to the study design (longitudinal or cross-sectional).

The EPHPP was used to evaluate longitudinal studies and comprises six categories that examine selection bias, confounding variables, data collection methods/instruments, blinding, and dropouts. Based on predetermined criteria, each category was scored as “poor”, “fair”, or “good”, determining an overall score defined as “weak”, “moderate”, or “strong”. Two authors (C.F. and S.A.) independently assigned a score to each study, and disagreements were solved by a third and more experienced author (E.R.G.) based on this review’s objectives.

The NOS instrument was used to assess cross-sectional studies and aims to determine the risk of bias, depending on three main categories: (1) sample selection, (2) comparability or control of confounders, and (3) outcomes. Points are given in each category (represented by *). Studies present a high risk of bias when classified between zero and three points, a moderate risk of bias between four and six points, and if a score greater than seven points was obtained, a low risk of bias was classified. Each article can receive a total of ten points in this assessment. Two authors carried out this assessment independently (F.S., C.F.), and disagreements were solved by a third and more experienced author (E.R.G.).

## 3. Results

### 3.1. Study Selection

**Figure 1:**
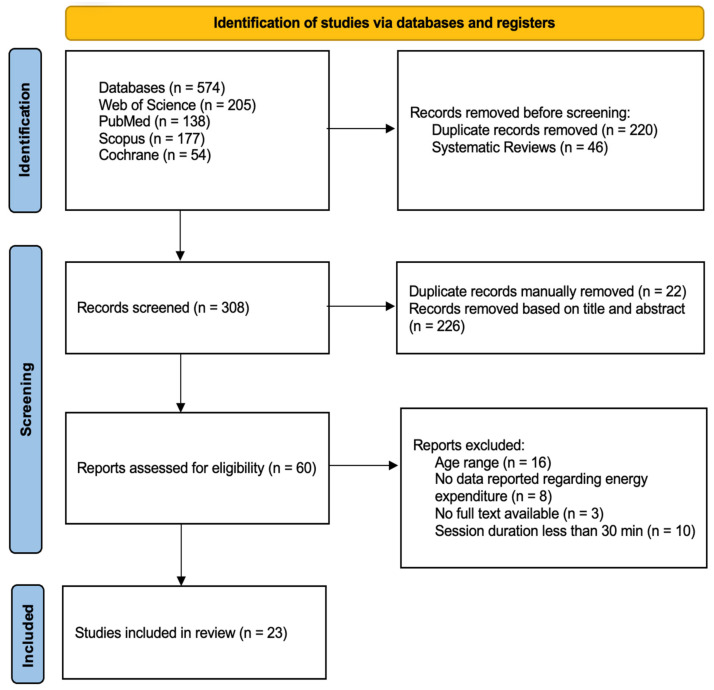
PRISMA flowchart of the studies’ selection.

Figure 1 resumes the flowchart of the study selection procedure. The initial search in four databases (Web of Science, PubMed, Scopus, and Cochrane) identified 574 manuscripts. Duplicates were automatically removed (n = 220), and systematic review articles were excluded (n = 46). From the 308 manuscripts remaining, 22 were manually removed as duplicate records, and 226 were eliminated based on title and abstract. The full-text analysis comprised 60 manuscripts, and 36 did not meet the inclusion criteria for the following reasons: age range (n = 16), no data reported regarding energy expenditure (n = 8), no full text available (n = 3), and the total AVGs’ session duration was less than 30 min (n = 10). Subsequently, 23 records were included in the analysis.

### 3.2. Risk of Bias Assessment

The detailed analysis of the study quality and risk of bias is summarized in the Supplementary File S1 (Tables S1 and S2). Ten studies were assessed using the EPHPP tool. Among those, two were classified as strong [24,25], six as moderate [10,26,27,28,29,30], and two as weak quality [31,32]. According to the EPHPP items, it was verified that most of the studies revealed no baseline differences between groups or accounted for at least 80% of the significant confounders (n = 8). All studies presented valid and reliable data collection instruments.

Thirteen studies were evaluated using the NOS. All of the studies scored greater than seven points and were, therefore, classified as having a low risk of bias. In the participants selection, only six studies obtained a score on the first question [33,34,35,36,37,38], as the sample was considered truly or somewhat representative of the target population. Regarding sample size, only two studies [37,39] obtained a score, since a justification for the sample size was presented. Most studies (n = 9) showed the inclusion or exclusion criteria, thus obtaining one point. Finally, all studies obtained two points, as the instruments used were considered valid. Regarding the comparability section, most studies (n = 11) received a maximum score of two points. In this parameter, even if the study did not have a control group, the top score was given when there was an adjustment or control of confounding factors, such as age, sex, or body weight. Finally, in the outcome section, all of the studies obtained the maximum score (three points), which clearly described the assessment and statistical analysis conducted.

### 3.3. Intervention Characteristics

Details regarding the study’s description, variables included, and main findings are presented in Table 1 and Table 2. Table 1 displays the cross-sectional studies (n = 13), while Table 2 resumes longitudinal interventions (n = 10).

Among all of the studies, 1439 children and adolescents were assessed. One study did not report the participants’ percentage regarding sex [35], two studies considered exclusively boys [37,39], and most of the overall participants were girls (n = 744). Three investigations have included only overweight participants [10,24,25], and three evaluated both overweight and normal-weight participants [35,39,43]. Participants’ ages ranged between 9 and 17 years; however, fewer studies considered adolescents aged at least 13 years (n = 9) [24,35,36,37,38,39,40,41,42].

In longitudinal studies, the shortest intervention duration was six weeks [28], while the longer one was deployed for 12 months [27]. The most common duration of the intervention was 12 weeks (n = 3) [24,26,31]. Regarding the methods implemented, in six studies, participants attended weekly sessions based on AVGs, ranging between 40 and 60 min [10,25,28,29,30,32], while in the other four studies, participants had access to AVGs and were incentivized to play them daily [24,26,27,31]. Most of the studies included a control group (CG) that did not engage in any AVGs (n = 9) [10,24,25,26,27,29,30,31,32], and one study only considered exclusively an intervention group (IG) [28]. Regarding cross-sectional studies, most included only one testing session (n = 9) [33,35,37,39,40,41,42,44,45], ranging between 30 and 60 min. 

**Figure 2:**
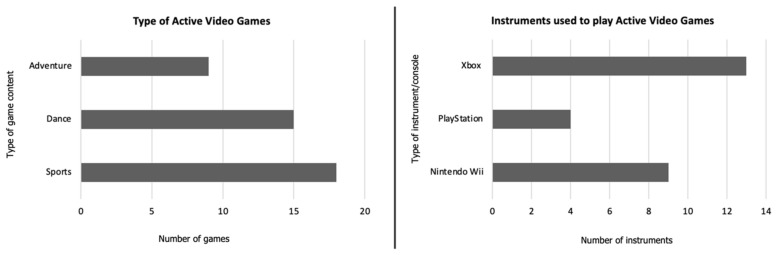
Types of AVGs and instruments used in the studies under analysis.

Figure 2 displays the types of AVGs selected, and the instruments used to play in the studies under analysis. Games based on sports (n = 18) and dance (n = 15) were the most common, and Xbox was the game console most used during the interventions/testing sessions (n = 13). It is of note that 15 studies have combined different types of AVGs [10,25,26,29,30,31,32,33,34,36,38,42,43,45], and four studies have implemented more than one game console [10,29,32,43].

### 3.4. Energy Expenditure during AVGs

Details concerning EE results are presented in the Supplementary File S2 (Tables S3 and S4).

*Longitudinal studies*

In longitudinal studies, the authors have relied mostly on accelerometers to measure PA levels. The results obtained were contradictory. In five interventions, no significant changes were seen in MVPA between the IG and CG [10,26,27,30,31]. In contrast, opposite results were described in four studies [24,25,28,29]. Additionally, one study reported increases in METs/day seen among the IG (baseline = 1.71 *±* 0.20; follow-up = 1.81 *±* 0.20), whereas the CG showed a decreased METs/day over time (baseline = 1.74 *±* 0.16; follow-up = 1.45 *±* 0.52) [32]. Interestingly, regarding MVPA and VPA, among the three studies that included overweight participants [10,24,25], only the intervention that combined AVGs with a program focused on nutritional knowledge has described significant improvements in participants’ MVPA and VPA (*p ≤* 0.05) at follow-up (week 16) [25]. 

*Cross-sectional studies*

In cross-sectional studies, indirect calorimetry was frequently used to assess EE (n = 11) [33,34,35,37,39,40,41,42,43,44,45]. Indeed, most of these studies have included a single testing session with a single group deployed in a laboratory context (n = 10) [33,34,36,37,38,40,41,42,44,45].

Among the investigations conducted, three have focused on comparing EE between overweight and normal-weight participants [35,39,43]. In two studies, no significant differences were seen between groups while playing AVGs in EE, and METs’ mean values [39,43]. Interestingly, in both of these studies, the authors reported that AVGs can reach moderate-intensity PA (3–6 METs), which may be relevant to developing and maintaining cardiorespiratory fitness [39,43]. 

The single-group analysis showed a significantly greater EE and HR during AVGs compared to rest conditions (*p* ≤ 0.05). In the investigations that included sex comparisons, boys tended to attain higher EE than girls [34,36,42,44]. Among the games selected, boxing consistently showed enhanced EE compared to other sports and dance games [38,40,42]. In three investigations, the data on EE and HR during AVGs were comparable to moderate-intensity PA [34,40,44].

Two studies aimed to compare EE between AVGs and nonactive games [33,37], and another two have evaluated the differences between playing modes (single vs. two-player mode) [38,45]. Consistently, AVGs elicited substantial increases in EE compared to nonactive gaming, with almost double the mean values of EE being reported during AVGs. Regarding playing mode, there was a tendency to achieve higher EE during the two-player mode compared to the single-player mode. 

## 4. Discussion

This systematic review aimed to provide an overall view of the current evidence on EE among adolescents while playing AVGs. According to the results, short-term AVG sessions (with at least 30 min of duration) elicited EE values comparable to moderate-intensity PA (3–6 METs). However, the analysis of intervention programs based on AVGs (with at least six weeks of duration) did not conclude significant changes in EE and PA profiles from the baseline to follow-up. AVGs based on sports and dance through the Xbox and Nintendo Wii were the most used during the interventions. Overall boys tended to achieve higher EE than girls.

Current guidelines for youth recommend at least 60 min per day of MVPA to achieve healthier lifestyles [1]. Among the 23 studies under analysis, five have described EE levels comparable to moderate-intensity PA (3–6 METs), which is consistent with the previous research conducted among youth and adult populations [7,46]. However, caution is needed in the interpretation of these results. Only one study has provided an AVG session of 60 min, while the other four focused on 30 min sessions, not meeting the recommended guidelines for PA. Besides, no distinguishment was made between PA intensity levels while playing the different AVGs, which would allow a better understanding of the impact derived from each game. This data is of great interest in designing AVG sessions, particularly in defining intensity thresholds based on the targeted physical fitness components. For instance, previous research has reported superior benefits for body composition and cardiorespiratory fitness outcomes of time spent in vigorous PA over moderate PA, which is likely due to the increased overall EE resulting from vigorous PA compared to a similar volume of moderate PA [47,48]. 

Meantime, both longitudinal and cross-sectional investigations were analyzed in the present study. To assess the efficacy of intervention programs based on AVGs, the authors have relied on accelerometry to quantify PA, and indirectly EE, at the baseline and follow-up. In fewer laboratory sessions, indirect calorimetry was frequently used to assess EE. Even though a diversity of protocols was implemented, the metrics used to interpret the results limits the comparison between studies. For instance, in cross-sectional approaches, while some authors used the MET [35,38,44] to report EE, others relied on results expressions based on kilocalories (kcal) [33,42,43,45] and joules (J) [34,36,37,39,40,41] to report their data. On the other hand, the number of sessions and their duration (ranging between 30 and 60 min) were also quite diverse between the investigations. Therefore, it is strongly recommended for future research to use standardized methods to allow a clearer comparison between studies. Based on the results of the present review, it seems that AVGs might be promoted between two and three times per week for at least 45 to 60 min.

In intervention programs, AVGs showed the potential to enhance PA, even if not with statistical effect. Interestingly, among the intervention programs that included overweight participants (n = 3), significant improvements between baseline and follow-up were only reported in the program that combined AVGs and a specific unit dedicated to nutritional knowledge [25]. Indeed, multidisciplinary approaches combining nutrition and PA at individual, family, and institutional levels, have been pointed out as crucial in the fight against youth obesity [49]. Therefore, although AVGs emerged as a potential source to reach moderate-intensity PA, which is relevant to promoting health-related physical components, effective intervention strategies should focus on behavioral changes [50], and combine the efforts of several actors (i.e., family, friends, school teachers) involved in the youngsters’ life. In this matter, Physical Education emerges as a crucial environment since it underlines the importance of PA. Indeed, future AVG interventions might be introduced in Physical Education classes allowing students to experience different ways to participate in PA. 

Most of the AVG sessions analyzed included sports (n = 18) and dance (n = 15) games. Among the studies that compared EE between AVGs, boxing games showed the highest EE levels (n = 3). On the other hand, in the investigations that included sex comparisons (n = 4), the authors reported that boys tended to attain superior EE than girls. In a previous meta-analysis, game type and player age were identified as significant moderators of the AVGs’ effects. Games including lower body and whole body movements produced more EE than upper body systems, and youth displayed higher EE than adults [46]. Regarding sex differences, a previous literature review also concluded that EE was higher for boys than girls [16], which might be related to the type of games used. As an example, dance activities are often more associated with girls’ preferences [9], while sports and boxing games could be more attractive to boys. Therefore, interventions designed for youth populations should consider gender, providing tailored programs that elicit the interest of the participants. Instant enjoyment and moderate challenge have been mentioned as key motivating elements for PA adherence [51] and, therefore, these aspects should be considered by AVGs designers and by sports and health professionals when defining intervention programs. 

The current study presents some limitations that should be highlighted. The diversity in the methods employed and the use of several EE outcomes limits the comparison between studies. Future research based on standardized methods would contribute to a more rigorous results comparison and support deeper conclusions. Moreover, several investigations included in this analysis were centered on one testing session (n = 9), which is influenced by the type and duration of the AVGs played in each study. Therefore, caution is recommended in the results’ interpretation. In longitudinal approaches, repetitive programs exclusively based on AVGs may lead to boredom among participants [27]. In addition, AVGs was only able to promote light to moderate PA levels, and opportunities to perform vigorous PA during youth have been strongly recommended in the literature [47]. 

Even though, there is evidence for the potential of AVGs to promote EE in youth, probably due to its attractiveness among youngsters, based on the current study, AVG programs might emerge as a different and complementary tool to traditional PA activities developed in schools and local communities. However, the lack of details concerning the comparison of EE between different AVG types and exploring the differences between boys’ and girls’ preferences justifies future research on the topic. Due to the contradictory findings identified, the current review also underlines the need to develop future investigations to examine the effects of a long-term intervention including AVGs on youngsters’ PA profiles.

## 5. Conclusions

The results of the present review suggest AVGs as a potential source of EE among youngsters, particularly to induce light to moderate PA levels. However, the diversity of methods used in the studies analyzed limits the comparison of results and drawing generalized conclusions. Overall, there is still a lack of longitudinal data on the impact of AVGs on health-related components. Indeed, among the twenty-three studies included, nine were based on one testing session. Future research designed to examine the effect of a long-term intervention including AVGs on PA levels is still needed. Considering its attractiveness to the younger population, AVGs might emerge as a complementary strategy to traditional PA activities to avoid game repetition boredom considering its lack of ability to promote vigorous PA levels. 

## Figures and Tables

**Table 1:** Summary of the longitudinal studies’ description, variables included, and main findings.

Study	Purpose	Sample	Instruments Used to Assess EE	Active Video Games	Main Findings
Mhurchu et al. (2008) [31]	To evaluate the effect of AVGs on children’s PA levels.	20 children (12 boys) aged 12.0 ± 1.5 years IG: n = 10 CG: n = 10	Accelerometer (ActiGraph Model AM7164-2.2C)	IG: children had access to an AVGs package (EyeToy sports active games, and dance mats) for PlayStation 2; children were incentivized to substitute their usual non-AVGs play with AVGs play for 12 weeks. CG: children engaged in their daily routines.	The IG spent significantly less time playing inactive games compared to the CG; at weeks 6 and 12, CPM was higher in the IG than in the CG (+194 CPM and +48 CPM, respectively); no significant differences in MPA were seen between groups; boys were significantly more active than girls.
Baranowski et al. (2011) [26]	To examine whether children receiving AVGs spontaneously engage in more PA than those receiving an inactive video game.	78 children (42 boys) aged between 9 and 12 years IG: n = 41 CG: n = 37	Accelerometer (ActiGraph GT3X, Pensacola, FL, USA)	IG: accessed Active Life-Extreme Challenge, EA Sports Active, DDR, Wii Fit Plus, and Wii sports for Nintendo Wii console for 12 weeks. CG: had access to 5 inactive games that were popular at the time of the study.	Children with access to AVGs were not more active over 12 weeks than those with access to an inactive video game (LPA: 400.9 ± 80.7 min/day and 412.5 ± 73.1 min/day, respectively).
Maloney et al. (2012) [24]	To determine if DDR would boost PA among children.	64 overweight (30 boys) children aged between 9 and 17 years IG: n = 33 (20 boys) CG: n = 31 (10 boys)	Accelerometer (ActiGraph GT1M and GT3X, Pensacola, FL, USA)	IG: had access to DDR for PlayStation 2 for 12 weeks. CG: instructed to maintain their routine.	No significant differences in LPA over time in either group; decline of MVPA over time in both groups, although not significantly; decline in VPA in both groups over time, but the trend was for the IG to decline less.
Azevedo et al. (2014) [27]	To examine the effect of providing a dance mats system in public secondary schools on PA levels.	497 children (188 boys) aged between 11 and 13 years IG: n = 280 (111 boys) CG: n = 217 (78 boys)	Accelerometer (ActiGraph GT3X, Pensacola, FL, USA)	IG: had free access to the dance mats systems for 12 months. CG: engage in their usual daily activities without access to the dance mats.	MVPA was not significantly different between groups (IG = 55.9 ± 24.9 min/day and CG = 57.8 ± 22.2 min/day); significant decrease in LPA and an increase in sedentary time in IG compared to CG.
Trost et al. (2014) [25]	To assess the effects of AVGs on PA and weight loss in children included in a weight management program.	75 overweight or obese children (34 boys) aged between 8 and 12 years P + AG (Program + Active Gaming Intervention): n = 34 (15 boys) PO (Program Only): n = 41 (19 boys)	Accelerometer (ActiGraph GT3X and GT3X+, Pensacola, FL, USA)	P + AG: family-based treatment of childhood obesity plus a game console and motion capture device (Xbox and Kinect) with access to Kinect Adventures and Kinect Sports for 16 weeks (1 session per week of 60 min) PO: family-based treatment of childhood obesity for 16 weeks (1 session per week of 60 min).	Significant increases in MPA were observed in the P + AG group at weeks 8 and 16 (+6 min/day); in the PO group, MVPA levels declined at weeks 8 and 16; the P + AG group exhibited a significant increase in VPA at week 16, while VPA levels remained relatively unchanged at week 8 and then declined at week 16 among the PO group.
Chen et al. (2017) [28]	To explore the effects of AVGs feedback and playing experience on individuals’ MVPA and perceived enjoyment.	36 children (15 boys) aged 10.2 ± 0.4 years	Accelerometer (ActiGraph GT3X+, Pensacola, FL, USA)	Participants played AVGs (Zumba Kids and Just Dance Kids for Xbox 360) during the regular PE class time for approximately 40 min. This procedure occurred 3 times per week for 6 weeks.	Zumba Kids (24.0 + 12.3) generated a significant greater MVPA % than Just Dance Kids (18.2 + 9.9); for Zumba Kids, higher MVPA % was observed at the last two sessions (25.3 + 13.9) compared to the first two sessions (24.5 + 16.5); for Just Dance Kids, significant lower MVPA % was seen in the last two sessions (19.3 + 13.1) compared to the first two sessions (22.1 + 14.7).
Gao et al. (2019) [32]	To examine the effects of AVGs on children’s school-day EE and PA-related self-efficacy and social support.	81 children (42 boys) aged between 9 and 10 years IG: n = 36 (20 boys) CG: n = 45 (22 boys)	Accelerometer (ActiGraph GT3X+, Pensacola, FL, USA)	IG: 1 weekly 50 min AVG (Just Dance, Wii Fit, Gold’s Gym Cardio Workout, and Kinect Sports for Xbox or Nintendo Wii) intervention beyond PE classes for 9 months. CG: children did not engage in any AVGs play or other structured school-based PA programs beyond PE.	The IG had increased METs/day (+0.10 METs), whereas the CG showed decreased METs/day over time.
Ye et al. (2019) [29]	To assess the effects of a school-based exergaming intervention on children’s objectively measured PA and CRF.	81 children (42 boys) aged between 9 and 10 years IG: n = 36 (20 boys) CG: n = 45 (22 boys)	Accelerometer (ActiGraph GT3X+, Pensacola, FL, USA)	IG: 1 weekly 50 min AVG (Just Dance, Wii Fit, Gold’s Gym Cardio Workout, and Kinect Sports for Xbox or Nintendo Wii) intervention beyond PE classes for 8 months. CG: children did not engage in any AVGs play nor any other structured school-based PA programs beyond PE.	The IG increased MVPA over time (+12.7 min), while the CG experienced a slight improvement (+2.3 min); CG showed an increased LPA over time (+15.3 min), while the IG presented a slight decrease (5.1 min); both groups decreased SB during the intervention.
Liang et al. (2020) [30]	To explore the potential of a school-based AVGs intervention on SB, PA, body composition, and psychosocial factors among children.	87 children (54 boys) aged between 9 and 12 years IG: n = 30 (24 boys) CG: n = 57 (30 boys)	Accelerometer (ActiGraph GT3X and GT3X+, Pensacola, FL, USA)	IG: attended two after-school AVGs (Kinect Adventures and Kinect Sports for Xbox) classes per week for 8 weeks; each session had a 60 min duration. CG: children who were not participating in exercise-based extracurricular activities during the intervention period.	During after-school time, a significant difference was observed between groups in SB (-23.5 min), LPA (+25.0 min) and CPM (+109.4), favoring the IG; although increased MVPA was observed in the IG at week 8, the comparison between groups was not substantial.
Comeras-Chueca et al. (2022) [10]	To examine the influence of an AVGs intervention combined with multicomponent exercise on muscular fitness, PA, and motor skills.	29 (16 boys) overweight or obese children aged between 9 and 12 years IG: n = 21 CG: n = 8	Accelerometer (GENEActiv, ActivInsights Ltd., Kimbolton, Cambridgeshire, UK)	IG: 3 sessions per week (60 min each) of AVGs together with multicomponent exercises performed between the AVGs, for 5 months. AVGs included Kinect Adventures, Kinect Sports, Wii Sports, Just Dance, Mario and Sonic at the Olympic Games, DDR, and the BKOOL interactive cycling simulator connected to a tablet. CG: children who were not included in the intervention.	The IG showed a significant reduction of SB, whereas no changes were observed in the CG; LPA significantly increased in the IG (+12.8 min/day); no significant differences were seen in MVPA in both groups.

EE (energy expenditure), AVGs (active video games), PA (physical activity), IG (intervention group), CG (control group), CPM (count per minute), DDR (Dance Dance Revolution), LPA (light PA), MVPA (moderate-to-vigorous PA), VPA (vigorous PA), PE (Physical Education), MET (metabolic equivalent), CRF (cardiorespiratory fitness), and SB (sedentary behavior).

**Table 2:** Summary of the cross-sectional studies description, variables included, and main findings.

Study	Purpose	Sample (Age Presented by M ± SD)	Instruments Used to Assess EE	Active Video Games	Main Findings
Maddison et al. (2007) [33]	To quantify the EE and PA while playing AVGs and nonactive video games	21 children (10 boys) aged 12.4 ± 1.1 years	HR (Polar Accurex, Kempele Finland); Accelerometer (Actigraph Model AM7164-2.2C); Indirect calorimetry (MetaMax3B, Cortex, Biophysik, Leipzig, Germany)	EyeToy Knockout, Homerun, Groove, AntiGrav, and Dance UK (PlayStation 2) for 1 session of 50 min.	Significant increase in EE, HR, and PA count while playing AVGs compared with rest and nonactive gaming; no significant differences based on sex.
Graves et al. (2008) [40]	To examine the contribution of upper limb and total body movement to adolescents’ EE whilst playing AVGs.	13 children (7 boys) aged 15.1 ± 1.4 years	Accelerometer (ActiGraph GT1M, Pensacola, FL, USA); Indirect calorimetry (MetaMax3B, Cortex, Biophysik, Leipzig, Germany)	Bowling, tennis, and boxing (Wii Sports) for 1 session of 60 min.	EE and HR were significantly greater in AVGs compared to sedentary gaming; boxing AVGs showed enhanced EE and HR; girls’ HR was significantly greater than boys during AVGs.
Graf et al. (2009) [34]	To compare EE rates in children playing AVGs and treadmill walking.	23 children (14 boys) aged 11.9 ± 1.2 years	Indirect calorimetry (Ultima CardiO2, Medgraphics, St. Paul, MN, USA); HR (Polar, PolarElectro, Helsinki, Finland); Accelerometer (StepWatch 3, OrthoCare Innovations, Mountlake, Terrace, WA, USA)	DDR, bowling, and boxing (Nintendo Wii) for 2 sessions of 30 min each.	EE and HR during AVGs were comparable to moderate-intensity walking (4.2–5.7 km/h); boys EE values were superior to girls while playing DDR and bowling.
Graves et al. (2010) [41]	To evaluate the physiological cost (HR and EE) and enjoyment while playing AVGs compared to nonactive gaming.	14 adolescents (10 boys) aged 15.8 years	Indirect calorimetry (MetaMax3B, Biophysik, Leipzig, Germany)	Yoga, muscle conditioning, balance, and aerobics Wii Fit (Nintendo Wii) for 1 session of 30 min.	EE and HR in AVGs were greater than nonactive gaming; Wii aerobics elicited moderate intensity activity in adolescents; Wii Fit was described as an enjoyable game.
Smallwood et al. (2012) [42]	To evaluate the physiologic responses and EE of AVGs.	18 children (10 boys) aged 13.4 ± 1.2 years	Indirect calorimetry (Cosmed K4b2)	Dance Central and Kinect Sports Boxing (Kinect Xbox 360) for 1 session of 30 min.	Significantly higher HR and EE during AVGs compared to nonactive video games; EE was higher for boys during Sports Boxing.
O’Donovan et al. (2013) [35]	To measure the energy cost of playing AVGs in OWC and NWC.	60 children OWC: 30 children aged 12.0 ± 3.0 years NWC: 30 children aged 12.0 ± 3.0 years	Indirect calorimetry (Oxycon Mobile, Viasys Healthcare Hoechberg, Germany); HR (Polar)	Both groups played Boxing and Free Jogging (Nintendo Wii) for 1 session of 30 min.	While playing Wii Fit Free Jogging, the OWC showed significantly less EE than their healthy peers; playing boxing was a light intensity activity for both groups, while jogging was a moderate intensity activity.
Rosenberg et al. (2013) [36]	To measure EE during AVGs bouts.	47 children (28 boys) aged between 10 and 15 years.	HR and EE (Actiheart monitor)	Kinect Adventures, Kinect Sports, Motion Sports Adrenaline, Sonic Free Riders, Just Dance 3 and Virtua Tennis 4 (Xbox 360) for 4 sessions of 45 min each.	EE between AVGs was similar; boys EE was higher than girls within each AVG and across the four gaming sessions.
Verhoeven et al. (2015) [38]	To examine whether children’s EE and game enjoyment are higher when AVGs are played in a two-player mode than in a single-player mode.	43 children (21 boys) aged 13.0 ± 0.88 years	Activity sensor (SenseWear 3 Pro armband, Bodymedia Inc., Pittsburgh, PA, USA)	Boxing, bowling, tennis, baseball, golf, and dancing games (Xbox 360) for 4 sessions of 60 min. The games were played in single player and two-player mode.	EE was higher in two-player mode when compared to single-player; no sex differences were found; boxing game showed the highest EE; no differences in game enjoyment occurred between playing modes.
Gribbon et al. (2015) [37]	To examine the acute effects of AVGs on energy intake and EE.	26 boys aged 14.5 ± 1.4 years	Indirect calorimetry (Cosmed K4b2); accelerometry (Actical, Philips Respironics)	Comparison of three experimental conditions: resting in a seated position, seated video game play (Xbox 360), and AVG (Kinect Adventures on Xbox 360) for 1 session of 60 min.	EE was significantly higher during AVGs conditions than in the resting and seated video games conditions.
Lau et al. (2015) [43]	To examine whether AVGs could help children reach the PA recommendations and CRF regarding exercise intensity.	21 children (17 boys) OWC: 8 children aged 10.4 ± 1.0 years NWC: 13 children aged 10.5 ± 0.8 years	Indirect calorimetry (MetaMax3B, Cortex Biophysik, Leipzig, Germany); HR (Polar, Lake Success, NY, USA); RPE.	Both groups played I-Dong running, Obstacle Course, I-Dong, Table Tennis, I-Dong Dancing, and Rhythm Kung-Fu (Nintendo Wii and PlayStation 3) for 2 sessions of 30 min each.	No significant differences between groups in absolute EE, VO_2_, HR, and METs during AVGs; AVGs can be used to reach the recommended intensity for developing and maintaining CRF.
Chaput et al. (2016) [39]	To examine EE among NWC and OWC while playing AVGs.	31 boys OWC: 19 boys aged 14.5 ± 0.8 years NWC: 12 boys aged 13.5 ± 1.7 years	Indirect calorimeter (Cosmed K4b2)	Both groups played Kinect Sport Boxing (Xbox 360) for 1 session of 60 min.	Significantly higher EE was recorded in the OWC but not when corrected for body composition; maximal HR during AVGs was significantly higher in lean adolescents; time spent between 3 and 6 METs was not different between groups.
McNarry et al. (2016) [44]	To investigate the relative intensity of AVGs in children.	34 children (20 boys) aged 10.8 ± 1.0 years	Indirect calorimetry (MetaMax3B, Cortex, Biophysik, Leipzig, Germany); HR (Polar, Kempele, Finland).	River Rush and Reflex Ridge (Xbox 360) for 1 session of 30 min.	AVGs elicited moderate intensity PA (3.0 METs); over a third of participants achieved vigorous intensity PA (6.0 METs); boys attained higher EE than girls.
Barkman et al. (2016) [45]	To compare the EE while playing single- vs. multiplayer mode of AVGs.	40 adolescents (26 boys) aged 11.0 ± 0.9 years	Indirect calorimetry (Oxycon Mobile, Cardinal Health, Yorba, Linda, CA, USA).	Kinect Adventures Reflex Ridge, Just Dance 3, Wipeout, and Kinect Sports Boxing (Xbox 360) for 1 session (60 min).	Higher EE was attained during multiplayer mode than single-player mode.

M ± SD (mean EE ± standard deviation), (energy expenditure), PA (physical activity), AVGs (active video games), HR (heart rate), DDR (Dance Dance Revolution), OWC (overweight children), NWC (normal-weight children), MET (metabolic equivalent), and CRF (cardiorespiratory fitness).

## Supplementary Materials

The following supporting information can be downloaded at: https://www.mdpi.com/article/10.3390/sports12020039/s1; Figure S1: PRISMA checklist; Table S1: Longitudinal studies methodological quality assessment using the Effective Public Health Practice Project; Table S2: Cross-sectional studies methodological assessment using the Newcastle-Ottawa Scale; Table S3: Descriptive statistics for energy expenditure among the longitudinal studies included in the analysis; Table S4: Descriptive statistics for energy expenditure among the cross-sectional studies included in the analysis.
