# Supplementary material for: Estimated Energy Expenditure in Youth While Playing Active Video Games: A Systematic Review
Source: Sports (Basel). 2024 Jan 25;12(2):39. doi: 10.3390/sports12020039 (PMC10892794; doi:10.3390/sports12020039)
Supplement: Supplementary file 1 [file sports-12-00039-s001.zip › 02_cf_a_SUPPLEMENTARY FILE 1_Table S1 and S2_QUALITY ASSESSMENT_EPHPP & NOS SCALE.pdf]

Table S1. Longitudinal studies methodological quality assessment using the Effective Public Health Practice Project.

Study	Selection bias	Design	Confounders	Blinding	Data collection methods	Withdrawals and drop-outs	Final score
Mhurchu et al. (2008) [1]	Fair	Good	Poor	Poor	Good	Fair	Weak
Baranowski et al. (2011) [2]	Fair	Good	Good	Poor	Good	Good	Moderate
Maloney et al. (2012) [3]	Fair	Good	Fair	Fair	Good	Good	Strong
Azevedo et al. (2014) [4]	Strong	Fair	Fair	Poor	Good	Good	Moderate
Trost et al. (2014) [5]	Good	Good	Good	Fair	Good	Good	Strong
Chen et al. (2017) [6]	Good	Fair	Good	Poor	Good	Good	Moderate
Gao et al. (2019) [7]	Good	Fair	Poor	Poor	Good	Good	Weak
Ye et al. (2019) [8]	Fair	Fair	Good	Poor	Good	Fair	Moderate
Liang et al. (2020) [9]	Fair	Good	Good	Poor	Good	Good	Moderate
Comeras-Chueca et al. (2022) [10]	Good	Good	Good	Poor	Good	Good	Moderate

Table S2. Cross-sectional studies methodological quality assessment using the Newcastle-Ottawa Scale.

Study	Selection				Comparability		Outcome		Total
	1	2	3	4	1a	1b	1	2	
Maddison et al. (2007) [11]	*		*	**	*		**	*	8*
Graves et al. (2008) [12]	*			**	*	*	**	*	8*
Graf et al. (2009) [13]				**	*	*	**	*	7*
Graves et al. (2010) [14]				**	*	*	**	*	7*
Smallwood et al. (2012) [15]				**	*	*	**	*	7*
O'Donovan et al. (2013) [16]	*		*	**	*	*	**	*	9*
Rosenberg et al. (2013) [17]	*		*	**	*	*	**	*	9*
Verhoeven et al. (2015) [18]		*	*	**	*	*	**	*	9*
Gribbon et al. (2015) [19]	*	*	*	**	*	*	**	*	10*
Lau et al. (2015) [20]			*	**	*		**	*	7*
Chaput et al. (2016) [21]	*		*	**	*	*	**	*	9*
McNarry et al. (2016) [22]			*	**	*	*	**	*	8*
Barkman et al. (2016) [23]			*	**	*	*	**	*	8*

References

1. Mhurchu, C.N.; Maddison, R.; Jiang, Y.; Jull, A.; Prapavessis, H.; Rodgers, A. Couch potatoes to jumping beans: A pilot study of the effect of active video games on physical activity in children. *International Journal of Behavioral Nutrition and Physical Activity* **2008**, *5*, 8. doi:10.1186/1479-5868-5-8.
2. Baranowski, T.; Abdelsamad, D.; Baranowski, J.; O'Connor, T.M.; Thompson, D.; Barnett, A.; Cerin, E.; Chen, T.A. Impact of an Active Video Game on Healthy Children's Physical Activity. *Pediatrics* **2012**, *129*, E636-E642, doi:10.1542/peds.2011-2050.
3. Maloney, A.E.; Threlkeld, K.A.; Cook, W.L. Comparative Effectiveness of a 12-Week Physical Activity Intervention for Overweight and Obese Youth: Exergaming with "Dance Dance Revolution". *Games for Health Journal* **2012**, *1*, 96-103. doi:10.1089/g4h.2011.0009.
4. Azevedo, L.B.; Watson, D.B.; Haighton, C.; Adams, J. The effect of dance mat exergaming systems on physical activity and health - related outcomes in secondary schools: results from a natural experiment. *Bmc Public Health* **2014**, *14*, 951. doi:10.1186/1471-2458-14-951.
5. Trost, S.G.; Sundal, D.; Foster, G.D.; Lent, M.R.; Vojta, D. Effects of a pediatric weight management program with and without active video games: a randomized trial. *JAMA pediatrics* **2014**, *168*, 407-413.
6. Chen, H.; Sun, H. The Effects of Active Videogame Feedback and Practicing Experience on Children's Physical Activity Intensity and Enjoyment. *Games Health J* **2017**, *6*, 200-204, doi:10.1089/g4h.2017.0027.
7. Gao, Z.; Pope, Z.C.; Lee, J.E.; Quan, M.H. Effects of Active Video Games on Children's Psychosocial Beliefs and School Day Energy Expenditure. *Journal of Clinical Medicine* **2019**, *8*, doi:10.3390/jcm8091268.
8. Ye, S.; Pope, Z.C.; Lee, J.E.; Gao, Z. Effects of school-based exergaming on urban children’s physical activity and cardiorespiratory fitness: A quasi-experimental study. *International journal of environmental research and public health* **2019**, *16*, 4080.
9. Liang, Y.; Lau, P.W.C.; Jiang, Y.; Maddison, R. Getting Active with Active Video Games: A Quasi-Experimental Study. *Int J Environ Res Public Health* **2020**, *17*, doi:10.3390/ijerph17217984.
10. Comeras-Chueca, C.; Villalba-Heredia, L.; Perez-Lasierra, J.L.; Marín-Puyalto, J.; Lozano-Berges, G.; Matute-Llorente, Á.; Vicente-Rodríguez, G.; Gonzalez-Aguero, A.; Casajús, J.A. Active video games improve muscular fitness and motor skills in children with overweight or obesity. *International Journal of Environmental Research and Public Health* **2022**, *19*, 2642.
11. Maddison, R.; Mhurchu, C.N.; Jull, A.; Jiang, Y.; Prapavessis, H.; Rodgers, A. Energy expended playing video console games: An opportunity to increase children's physical activity? *Pediatric Exercise Science* **2007**, *19*, 334-343, doi:10.1123/pes.19.3.334.
12. Graves, L.E.F.; Ridgers, N.D.; Stratton, G. The contribution of upper limb and total body movement to adolescents' energy expenditure whilst playing Nintendo Wii. *European Journal of Applied Physiology* **2008**, *104*, 617-623, doi:10.1007/s00421-008-0813-8.
13. Graf, D.L.; Pratt, L.V.; Hester, C.N.; Short, K.R. Playing Active Video Games Increases Energy Expenditure in Children. *Pediatrics* **2009**, *124*, 534-540, doi:10.1542/peds.2008-2851.

14. Graves, L.E.; Ridgers, N.D.; Williams, K.; Stratton, G.; Atkinson, G.; Cable, N.T. The physiological cost and enjoyment of Wii Fit in adolescents, young adults, and older adults. *J Phys Act Health* **2010**, *7*, 393-401, doi:10.1123/jpah.7.3.393.
15. Smallwood, S.R.; Morris, M.M.; Fallows, S.J.; Buckley, J.P. Physiologic Responses and Energy Expenditure of Kinect Active Video Game Play in Schoolchildren. *Archives of Pediatrics & Adolescent Medicine* **2012**, *166*, 1005-1009, doi:10.1001/archpediatrics.2012.1271.
16. O'Donovan, C.; Roche, E.F.; Hussey, J. The energy cost of playing active video games in children with obesity and children of a healthy weight. *Pediatric Obesity* **2014**, *9*, 310-317, doi:10.1111/j.2047-6310.2013.00172.x.
17. Rosenberg, M.; Lay, B.; Lee, M.; Derbyshire, A.; Kur, J.; Ferguson, R.; Maitland, C.; Mills, A.; Davies, C.; Pratt, I.S.; et al. New-Generation Active Videogaming Maintains Energy Expenditure in Children Across Repeated Bouts. *Games for Health Journal* **2013**, *2*, 274-279, doi:10.1089/g4h.2013.0037.
18. Verhoeven, K.; Abeele, V.V.; Gers, B.; Seghers, J. Energy Expenditure During Xbox Kinect Play in Early Adolescents: The Relationship with Player Mode and Game Enjoyment. *Games for Health Journal* **2015**, *4*, 444-451, doi:10.1089/g4h.2014.0106.
19. Gribbon, A.; McNeil, J.; Jay, O.; Tremblay, M.S.; Chaput, J.P. Active video games and energy balance in male adolescents: a randomized crossover trial. *American Journal of Clinical Nutrition* **2015**, *101*, 1126-1134, doi:10.3945/ajcn.114.105528.
20. Lau, P.W.C.; Liang, Y.; Lau, E.Y.; Choi, C.R.; Kim, C.G.; Shin, M.S. Evaluating physical and perceptual responses to exergames in chinese children. *International Journal of Environmental Research and Public Health* **2015**, *12*, 4018-4030, doi:10.3390/ijerph120404018.
21. Chaput, J.P.; Genin, P.M.; Le Moel, B.; Pereira, B.; Boirie, Y.; Duclos, M.; Thivel, D. Lean adolescents achieve higher intensities but not higher energy expenditure while playing active video games compared with obese ones. *Pediatric Obesity* **2016**, *11*, 102-106, doi:10.1111/ijpo.12027.
22. McNarry, M.A.; Mackintosh, K.A. Investigating the Relative Exercise Intensity of Exergames in Prepubertal Children. *Games for Health Journal* **2016**, *5*, 135-140, doi:10.1089/g4h.2015.0094.
23. Barkman, J.; Pfeiffer, K.; Diltz, A.; Peng, W. Examining Energy Expenditure in Youth Using XBOX Kinect: Differences by Player Mode. *Journal of Physical Activity & Health* **2016**, *13*, S41-S43, doi:10.1123/jpah.2016-0016.
