# Supplementary material for: Estimated Energy Expenditure in Youth While Playing Active Video Games: A Systematic Review
Source: Sports (Basel). 2024 Jan 25;12(2):39. doi: 10.3390/sports12020039 (PMC10892794; doi:10.3390/sports12020039)
Supplement: Supplementary file 1 [file sports-12-00039-s001.zip › 02_cf_a_SUPPLEMENTARY FILE 2_Table S3 and S4_EE VARIABLES ALL STUDIES.pdf]

Table S3. Descriptive statistics for energy expenditure among the longitudinal studies included in the analysis.

Study	EE results (M ± SD)
Mhurchu et al. (2008) [1]	MPA (baseline): IG = 97 ± 48 min/day; CG = 69 ± 23 min/day<br>VPA (baseline): IG = 7 ± 8 min/day; CG = 7 ± 7 min/day<br>*No descriptive data at follow-up
Baranowski et al. (2011) [2]	LPA (baseline): IG = 388.4 ± 69.4 min/day; CG = 399.6 ± 64.0 min/day<br>LPA (week 12): IG = 400.9 ± 80.7 min/day; CG = 412.5 ± 73.1 min/day<br>MVPA (baseline): IG = 27.4 ± 19.3 min/day; CG = 25.1 ± 15.0 min/day<br>MVPA (week 12): IG = 27.9 ± 14.3 min/day; CG = 27.6 ± 15.0 min/day
Maloney et al. (2012) [3]	Steps (baseline): IG = 6297 ± 4914; CG = 6838 ± 1766<br>Steps (follow-up): IG = 4914 ± 1443; CG = 6685 ± 1969<br>LPA (baseline): IG = 128.7 ± 33.2 min/day; CG = 127.5 ± 29.3 min/day<br>LPA (follow-up): IG = 126.0 ± 24.2 min/day; CG = 130.0 ± 35.7 min/day<br>MVPA (baseline): IG = 65.0 ± 22.3 min/day; CG = 71.3 ± 30.3 min/day<br>MVPA (follow-up): IG = 62.8 ± 25.7 min/day; CG = 62.1 ± 25.5 min/day<br>VPA (baseline): IG = 10.0 ± 7.7 min/day; CG = 17.0 ± 11.7 min/day<br>VPA (follow-up): IG = 9.3 ± 9.6 min/day; CG = 11.8 ± 8.7 min/day
Azevedo et al. (2014) [4]	LPA (baseline): IG = 236.7 ± 44.1 min/day; CG = 240.3 ± 47.3 min/day<br>LPA (follow-up): IG = 211.2 ± 41.0 min/day; CG = 232.4 ± 46.9 min/day<br>MVPA (baseline): IG = 61.3 ± 23.8 min/day; CG = 55.7 ± 22.0 min/day<br>MVPA (follow-up): IG = 55.9 ± 24.9 min/day; CG = 57.8 ± 22.2 min/day
Trost et al. (2014) [5]	MPA (baseline): Program + Active Gaming = 25.2 ± 2.6 min/day; Program Only = 27.0 ± 2.3 min/day<br>MPA (week 16): Program + Active Gaming = 31.3 ± 2.5 min/day; Program Only = 26.9 ± 2.3 min/day<br>VPA (baseline): Program + Active Gaming = 4.4 ± 0.7 min/day; Program Only = 4.9 ± 0.7 min/day<br>VPA (week 16): Program + Active Gaming = 6.4 ± 0.7 min/day; Program Only = 4.9 ± 0.7 min/day
Chen et al. (2017) [6]	MVPA (%) ranged between 18.2 ± 9.9 % (Just Dance Kids) and 24.0 ± 12.3 % (Zumba Kids)
Gao et al. (2019) [7]	METs (baseline): IG = 1.71 ± 0.20; CG = 1.74 ± 0.16<br>METs (9th month): IG = 1.81 ± 0.20; CG = 1.45 ± 0.52
Ye et al. (2019) [8]	LPA (baseline): IG = 266.9 ± 63.5 min/day; CG = 251.3 ± 46.9 min/day<br>LPA (8th month): IG = 261.8 ± 49.2 min/day; CG = 266.6 ± 51.6 min/day<br>MVPA (baseline): IG = 76.7 ± 22.0 min/day; CG = 61.8 ± 17.2 min/day<br>MVPA (8th month): IG = 89.4 ± 29.4 min/day; CG = 64.1 ± 18.0 min/day
Liang et al. (2020) [9]	LPA (baseline): IG = 107.8 ± 60.1 min/day; CG = 113.3 ± 45.6 min/day<br>LPA (week 8): IG = 101.6 ± 60.7 min/day; CG = 81.0 ± 47.8 min/day<br>MVPA (baseline): IG = 8.4 ± 5.7 min/day; CG = 10.5 ± 7.4 min/day<br>MVPA (week 8): IG = 8.8 ± 5.8 min/day; CG = 7.1 ± 6.7 min/day
Comeras-Chueca et al. (2022) [10]	LPA (baseline): IG = 71.3 ± 20.3 min/day; CG = 77.9 ± 30.9 min/day<br>LPA (5th month): IG = 84.1 ± 19.2 min/day; CG = 76.1 ± 19.0 min/day<br>MVPA (baseline): IG = 48.4 ± 27.4 min/day; CG = 36.6 ± 17.8 min/day<br>MVPA (5th month): IG = 57.1 ± 32.3 min/day; CG = 41.8 ± 17.7 min/day

EE (energy expenditure), M ± SD (mean ± standard deviation), PA (physical activity), IG (intervention group), CG (control group), MPA (moderate PA), LPA (light PA), VPA (vigorous PA), MVPA (moderate-to-vigorous PA), MET (metabolic equivalent)

Table S4. Descriptive statistics for energy expenditure among the cross-sectional studies included in the analysis.

Study	EE results (M ± SD)
Maddison et al. (2007) [11]	AVG: EE ranged between 2.9 ± 0.7 kcal/min to 6.5 ± 1.7 kcal/min<br>Nonactive videogames: EE ranged between 1.3 ± 0.2 kcal/min and 1.6 ± 0.2 kcal/min
Graves et al. (2008) [12]	AVG: EE ranged between 182.1 ± 41.3 J/kg/min and 267.2 ± 115.8 J/kg/min; HR ranged between 103.2 ± 16.7 and 136.7 ± 24.5 bpm
Graf et al. (2009) [13]	AVG: EE mean values were 11 and 13 kJ/min for DDR1 and DDR2, respectively; 12 kJ/min for boxing, and 8 kJ/min for bowling
Graves et al. (2010) [14]	AVG: EE ranged between 188.2 ± 31.0 J/kg/min and 236.8 ± 36.4 J/kg/min
Smallwood et al. (2012) [15]	AVG: EE ranged between 3.0 ± 1.0 kcal/min and 4.4 ± 1.6 kcal/min
O'Donovan et al. (2013) [16]	OWC (AVG): EE ranged between 2.72 ± 0.68 and 5.35 ± 1.52 METs<br>NWC (AVG): EE ranged between 2.99 ± 1.03 and 4.87 ± 1.40 METs
Rosenberg et al. (2013) [17]	AVG: EE ranged between 263 ± 83 J/kg/min and 286 ± 79 J/kg/min
Verhoeven et al. (2015) [18]	OWC (AVG): EE = 1547.93 ± 186.9 KJ per 60 min; METs = 3.49 ± 0.66; HR = 119.67 ± 13.35 bpm<br>NWC (AVG): EE = 1113.06 ± 474.48 (kJ per 60 min; METs = 4.55 ± 1.04; HR = 126.14 ± 29.89 bpm
Gribbon et al. (2015) [19]	Single-player mode: EE ranged between 2.87 ± 0.92 and 6.27 ± 1.37 METs<br>Two-player mode: EE ranged between 3.21 ± 0.50 and 7.04 ± 1.59 METs
Lau et al. (2015) [20]	AVG: EE = 1084 kJ<br>Seated video games: EE = 440 kJ
Chaput et al. (2016) [21]	OWC (AVG): EE ranged between 2.22 ± 0.45 and 5.23 ± 1.81 kcal/min<br>NWC (AVG): EE ranged between 1.95 ± 0.71 and 5.08 ± 1.15 kcal/min
McNarry et al. (2016) [22]	ACG: EE ranged between 5.5 ± 1.4 and 5.7 ± 1.5 METs
Barkman et al. (2016) [23]	Single-player mode: EE ranged between 3.1 ± 0.8 and 3.9 ± 1.0 kcal/min<br>Multiple player mode: EE ranged between 3.1 ± 0.9 and 4.1 ± 0.8 kcal/min

EE (energy expenditure); M ± SD (mean ± standard deviation), AVG (active video games), HR (heart rate), bpm (beats per minute), DDR (Dance Dance Revolution), OWC (overweight children), NWC (normal-weight children), MET (metabolic equivalent)

References

1. Mhurchu, C.N.; Maddison, R.; Jiang, Y.; Jull, A.; Prapavessis, H.; Rodgers, A. Couch potatoes to jumping beans: A pilot study of the effect of active video games on physical activity in children. *International Journal of Behavioral Nutrition and Physical Activity* **2008**, *5*, 8. doi:10.1186/1479-5868-5-8.
2. Baranowski, T.; Abdelsamad, D.; Baranowski, J.; O'Connor, T.M.; Thompson, D.; Barnett, A.; Cerin, E.; Chen, T.A. Impact of an Active Video Game on Healthy Children's Physical Activity. *Pediatrics* **2012**, *129*, E636-E642, doi:10.1542/peds.2011-2050.
3. Maloney, A.E.; Threlkeld, K.A.; Cook, W.L. Comparative Effectiveness of a 12-Week Physical Activity Intervention for Overweight and Obese Youth: Exergaming with "Dance Dance Revolution". *Games for Health Journal* **2012**, *1*, 96-103. doi:10.1089/g4h.2011.0009.
4. Azevedo, L.B.; Watson, D.B.; Haighton, C.; Adams, J. The effect of dance mat exergaming systems on physical activity and health - related outcomes in secondary schools: results from a natural experiment. *Bmc Public Health* **2014**, *14*, 951. doi:10.1186/1471-2458-14-951.
5. Trost, S.G.; Sundal, D.; Foster, G.D.; Lent, M.R.; Vojta, D. Effects of a pediatric weight management program with and without active video games: a randomized trial. *JAMA pediatrics* **2014**, *168*, 407-413.
6. Chen, H.; Sun, H. The Effects of Active Videogame Feedback and Practicing Experience on Children's Physical Activity Intensity and Enjoyment. *Games Health J* **2017**, *6*, 200-204, doi:10.1089/g4h.2017.0027.
7. Gao, Z.; Pope, Z.C.; Lee, J.E.; Quan, M.H. Effects of Active Video Games on Children's Psychosocial Beliefs and School Day Energy Expenditure. *Journal of Clinical Medicine* **2019**, *8*, doi:10.3390/jcm8091268.
8. Ye, S.; Pope, Z.C.; Lee, J.E.; Gao, Z. Effects of school-based exergaming on urban children’s physical activity and cardiorespiratory fitness: A quasi-experimental study. *International journal of environmental research and public health* **2019**, *16*, 4080.
9. Liang, Y.; Lau, P.W.C.; Jiang, Y.; Maddison, R. Getting Active with Active Video Games: A Quasi-Experimental Study. *Int J Environ Res Public Health* **2020**, *17*, doi:10.3390/ijerph17217984.
10. Comeras-Chueca, C.; Villalba-Heredia, L.; Perez-Lasierra, J.L.; Marín-Puyalto, J.; Lozano-Berges, G.; Matute-Llorente, Á.; Vicente-Rodríguez, G.; Gonzalez-Aguero, A.; Casajús, J.A. Active video games improve muscular fitness and motor skills in children with overweight or obesity. *International Journal of Environmental Research and Public Health* **2022**, *19*, 2642.
11. Maddison, R.; Mhurchu, C.N.; Jull, A.; Jiang, Y.; Prapavessis, H.; Rodgers, A. Energy expended playing video console games: An opportunity to increase children's physical activity? *Pediatric Exercise Science* **2007**, *19*, 334-343, doi:10.1123/pes.19.3.334.
12. Graves, L.E.F.; Ridgers, N.D.; Stratton, G. The contribution of upper limb and total body movement to adolescents' energy expenditure whilst playing Nintendo Wii. *European Journal of Applied Physiology* **2008**, *104*, 617-623, doi:10.1007/s00421-008-0813-8.
13. Graf, D.L.; Pratt, L.V.; Hester, C.N.; Short, K.R. Playing Active Video Games Increases Energy Expenditure in Children. *Pediatrics* **2009**, *124*, 534-540, doi:10.1542/peds.2008-2851.

14. Graves, L.E.; Ridgers, N.D.; Williams, K.; Stratton, G.; Atkinson, G.; Cable, N.T. The physiological cost and enjoyment of Wii Fit in adolescents, young adults, and older adults. *J Phys Act Health* **2010**, *7*, 393-401, doi:10.1123/jpah.7.3.393.
15. Smallwood, S.R.; Morris, M.M.; Fallows, S.J.; Buckley, J.P. Physiologic Responses and Energy Expenditure of Kinect Active Video Game Play in Schoolchildren. *Archives of Pediatrics & Adolescent Medicine* **2012**, *166*, 1005-1009, doi:10.1001/archpediatrics.2012.1271.
16. O'Donovan, C.; Roche, E.F.; Hussey, J. The energy cost of playing active video games in children with obesity and children of a healthy weight. *Pediatric Obesity* **2014**, *9*, 310-317, doi:10.1111/j.2047-6310.2013.00172.x.
17. Rosenberg, M.; Lay, B.; Lee, M.; Derbyshire, A.; Kur, J.; Ferguson, R.; Maitland, C.; Mills, A.; Davies, C.; Pratt, I.S.; et al. New-Generation Active Videogaming Maintains Energy Expenditure in Children Across Repeated Bouts. *Games for Health Journal* **2013**, *2*, 274-279, doi:10.1089/g4h.2013.0037.
18. Verhoeven, K.; Abeele, V.V.; Gers, B.; Seghers, J. Energy Expenditure During Xbox Kinect Play in Early Adolescents: The Relationship with Player Mode and Game Enjoyment. *Games for Health Journal* **2015**, *4*, 444-451, doi:10.1089/g4h.2014.0106.
19. Gribbon, A.; McNeil, J.; Jay, O.; Tremblay, M.S.; Chaput, J.P. Active video games and energy balance in male adolescents: a randomized crossover trial. *American Journal of Clinical Nutrition* **2015**, *101*, 1126-1134, doi:10.3945/ajcn.114.105528.
20. Lau, P.W.C.; Liang, Y.; Lau, E.Y.; Choi, C.R.; Kim, C.G.; Shin, M.S. Evaluating physical and perceptual responses to exergames in chinese children. *International Journal of Environmental Research and Public Health* **2015**, *12*, 4018-4030, doi:10.3390/ijerph120404018.
21. Chaput, J.P.; Genin, P.M.; Le Moel, B.; Pereira, B.; Boirie, Y.; Duclos, M.; Thivel, D. Lean adolescents achieve higher intensities but not higher energy expenditure while playing active video games compared with obese ones. *Pediatric Obesity* **2016**, *11*, 102-106, doi:10.1111/ijpo.12027.
22. McNarry, M.A.; Mackintosh, K.A. Investigating the Relative Exercise Intensity of Exergames in Prepubertal Children. *Games for Health Journal* **2016**, *5*, 135-140, doi:10.1089/g4h.2015.0094.
23. Barkman, J.; Pfeiffer, K.; Diltz, A.; Peng, W. Examining Energy Expenditure in Youth Using XBOX Kinect: Differences by Player Mode. *Journal of Physical Activity & Health* **2016**, *13*, S41-S43, doi:10.1123/jpah.2016-0016.
